# Maternal and perinatal death surveillance and response: a systematic review of qualitative studies

**DOI:** 10.2471/BLT.22.288703

**Published:** 2022-11-02

**Authors:** Merlin L Willcox, Immaculate A Okello, Alice Maidwell-Smith, Abera K Tura, Thomas van den Akker, Marian Knight

**Affiliations:** a School of Primary Care, Population Sciences and Medical Education, University of Southampton, Aldermoor Health Centre, Aldermoor Close, Southampton SO16 5SE, England.; b School of Nursing and Midwifery, Haramaya University, Harar, Ethiopia.; c Department of Obstetrics and Gynaecology, Leiden University Medical Centre, Leiden, Netherlands.; d National Perinatal Epidemiology Unit, University of Oxford, Oxford, England.

## Abstract

**Objective:**

To understand the experiences and perceptions of people implementing maternal and/or perinatal death surveillance and response in low- and middle-income countries, and the mechanisms by which this process can achieve its intended outcomes.

**Methods:**

In June 2022, we systematically searched seven databases for qualitative studies of stakeholders implementing maternal and/or perinatal death surveillance and response in low- and middle-income countries. Two reviewers independently screened articles and assessed their quality. We used thematic synthesis to derive descriptive themes and a realist approach to understand the context–mechanism–outcome configurations.

**Findings:**

Fifty-nine studies met the inclusion criteria. Good outcomes (improved quality of care or reduced mortality) were underpinned by a functional action cycle. Mechanisms for effective death surveillance and response included learning, vigilance and implementation of recommendations which motivated further engagement. The key context to enable effective death surveillance and response was a blame-free learning environment with good leadership. Inadequate outcomes (lack of improvement in care and mortality and discontinuation of death surveillance and response) resulted from a vicious cycle of under-reporting, inaccurate data, and inadequate review and recommendations, which led to demotivation and disengagement. Some harmful outcomes were reported, such as inappropriate referrals and worsened staff shortages, which resulted from a fear of negative consequences, including blame, disciplinary action or litigation.

**Conclusion:**

Conditions needed for effective maternal and/or perinatal death surveillance and response include: separation of the process from litigation and disciplinary procedures; comprehensive guidelines and training; adequate resources to implement recommendations; and supportive supervision to enable safe learning.

## Résumé

**Objectif:**

Comprendre les expériences et perceptions des individus chargés de mettre en œuvre la surveillance des décès maternels et périnatals et la riposte dans les pays à revenu faible et intermédiaire, ainsi que les mécanismes utilisés pour que ce processus atteigne ses objectifs.

**Méthodes:**

En juin 2022, nous avons analysé systématiquement sept bases de données à la recherche d'études qualitatives sur les intervenants responsables du processus de surveillance des décès maternels et périnatals et de la riposte dans les pays à revenu faible et intermédiaire. Deux réviseurs ont passé séparément les articles en revue afin d'évaluer leur qualité. Nous avons ensuite utilisé une synthèse thématique pour extraire des thèmes descriptifs et une approche réaliste permettant d'identifier les configurations contexte–mécanisme–résultat.

**Résultats:**

Nous avons inclus 59 études correspondant aux critères d'inclusion. Les résultats positifs (amélioration de la qualité des soins ou diminution de la mortalité) reposaient sur un cycle fonctionnel d'actions. Parmi les mécanismes favorisant la surveillance et la riposte contre les décès figuraient une expérience instructive, la vigilance et l'application de recommandations, ce qui motivait les acteurs à participer. Un environnement éducatif dépourvu de culpabilisation et bien dirigé offrait un contexte optimal pour garantir l'efficacité de la surveillance et de la riposte. Au contraire, les résultats insatisfaisants (absence d'amélioration des soins et de la mortalité, interruption du processus de surveillance et de riposte contre les décès) étaient liés à un cercle vicieux fait de sous-déclaration des décès, de renseignements inexacts, mais aussi d'analyses et de recommandations inadéquates, entraînant une démotivation et un manque d'engagement. Certains résultats néfastes ont également été rapportés, tels que des références inappropriées et une aggravation du manque de personnel, suscités par la crainte de conséquences négatives (blâmes, sanctions disciplinaires ou litiges).

**Conclusion:**

Plusieurs conditions sont requises pour assurer une surveillance et une riposte efficaces contre les décès maternels et/ou périnatals: séparer ce processus des procédures disciplinaires et des litiges; proposer un ensemble de formations et de lignes directrices; fournir les ressources nécessaires à la mise en œuvre des recommandations; et enfin, opter pour une supervision constructive, propice à un environnement éducatif sans danger.

## Resumen

**Objetivo:**

Comprender las experiencias y percepciones de las personas que implementan la vigilancia y la respuesta a la mortalidad materna o perinatal en los países de ingresos bajos y medios, y los mecanismos por los que este proceso puede alcanzar los resultados previstos.

**Métodos:**

En junio de 2022, se realizaron búsquedas sistemáticas en siete bases de datos para encontrar estudios cualitativos de las partes interesadas que implementan la vigilancia y la respuesta a la mortalidad materna o perinatal en países de ingresos bajos y medios. Dos revisores analizaron de forma independiente los artículos y evaluaron su calidad. Se utilizó la síntesis temática para derivar temas descriptivos y un enfoque realista para comprender las configuraciones de contexto, mecanismo y resultado.

**Resultados:**

Cincuenta y nueve estudios cumplieron los criterios de inclusión. Los resultados satisfactorios (mejora de la calidad de la atención o reducción de la mortalidad) se sustentaron en un ciclo de acción funcional. Los mecanismos para una vigilancia y respuesta eficaces a la mortalidad incluyeron el aprendizaje, la vigilancia y la aplicación de recomendaciones que motivaron un mayor compromiso. El contexto clave para hacer posible una vigilancia y respuesta eficaz a la mortalidad fue un entorno de aprendizaje libre de culpa con un buen liderazgo. Los resultados insuficientes (falta de mejora en la atención y la mortalidad e interrupción de la vigilancia y la respuesta a la mortalidad) fueron el resultado de un círculo vicioso de falta de notificación, datos inexactos y revisión y recomendaciones inadecuadas, que condujeron a la desmotivación y la falta de compromiso. Se notificaron algunos desenlaces perjudiciales, como las derivaciones incorrectas y una mayor falta de personal, que se debieron al miedo a las consecuencias negativas, como la culpa, las medidas disciplinarias o los litigios.

**Conclusión:**

Entre los requisitos necesarios para que la vigilancia y la respuesta a la mortalidad materna o perinatal sean eficaces se encuentran los siguientes: separación del proceso de los litigios y los procedimientos disciplinarios; directrices y formación exhaustivas; recursos adecuados para aplicar las recomendaciones; y una supervisión de apoyo que permita el aprendizaje seguro.

## ملخص

الغرض فهم تجارب وتصورات الأشخاص الذين يقومون بمراقبة وفيات الأمهات و/أو وفيات الفترة المحيطة بالولادة، والاستجابة لها في الدول ذات الدخل المنخفض والدخل المتوسط، والآليات التي يمكن لهذه العملية من خلالها تحقيق النتائج المرجوة.

الطريقة في يونيو/حزيران 2022، قمنا بالبحث بشكل منهجي في سبع قواعد بيانات للدراسات النوعية لأصحاب المصلحة الذين يقومون بتنفيذ مراقبة وفيات الأمهات و/أو وفيات الفترة المحيطة بالولادة، والاستجابة لها في الدول ذات الدخل المنخفض والدخل المتوسط. قام اثنان من المراجعين بشكل مستقل بفحص المقالات وتقييم جودتها. وقد استخدمنا التجميع الموضوعي لاشتقاق موضوعات وصفية، وأسلوب واقعي لفهم أوضاع السياق والآلية والنتائج.

النتائج استوفت تسع وخمسون دراسة معايير الاشتمال. كانت النتائج الجيدة (تحسين جودة الرعاية أو انخفاض معدل الوفيات) مدعومة بدورة عمل وظيفية. تضمنت آليات المراقبة الفعالة للوفيات والاستجابة لها، كل من التعلم واليقظة وتنفيذ التوصيات، وهو ما شجّع على مزيد من المشاركة. كان السياق الرئيسي لتمكين المراقبة الفعالة للوفيات والاستجابة لها هو بيئة تعليمية خالية من اللوم مع قيادة جيدة. نتجت النتائج غير الكافية (عدم وجود تحسن في الرعاية، والوفيات، وتوقّف مراقبة الوفيات والاستجابة لها) عن حلقة مفرغة من نقص الإبلاغ، والبيانات غير الدقيقة، والمراجعة والتوصيات غير الكافية، وهو ما أدى إلى تثبيط الهمة والانسحاب. تم الإبلاغ عن بعض النتائج الضارة، مثل الإحالات غير الملائمة، وحالات النقص المتدهور في الموظفين، والذي نتج عن الخوف من التبعات السلبية، بما في ذلك اللوم، أو الإجراءات التأديبية، أو التقاضي.

الاستنتاج تشمل الشروط المطلوبة للمراقبة الفعالة لوفيات الأمهات و/أو وفيات الفترة المحيطة بالولادة والاستجابة لها ما يلي: فصل العملية عن الإجراءات القضائية والتأديبية؛ الإرشادات الشاملة والتدريب؛ والموارد الكافية لتنفيذ التوصيات؛ والإشراف الداعم لتمكين التعلم الآمن.

## 摘要

**目的:**

旨在了解中低收入国家实施孕产妇和/或围产期死亡监测和响应的经验和相关人员的看法，以及该流程实现预期结果的机制。

**方法:**

在 2022 年 6 月，我们系统地搜索了七个定性研究数据中关于在中低收入国家实施孕产妇和/或围产期死亡监测和响应的利益相关者的数据。两名评审员对论文进行了独立筛选并评估了论文质量。我们将各个主题进行整合以获得描述性的主题，并使用实际可行的方法来了解情境-机制-结果的结构关系。

**结果:**

59 项研究符合纳入标准。周期性地采取功能性行动带来了积极结果（提高护理质量或降低死亡率）。有效的死亡监测和响应机制包括学习、保持警觉并执行可促进更多人员参与的建议。实现有效死亡监测和响应的关键条件是打造免于受责的学习环境和优秀的领导力。不充分的结果（不能改善护理质量和降低死亡率以及持续实施死亡监测和响应）源自漏报、数据不准确、审查和建议不足造成的恶性循环，这导致了消极怠工和工作疏离感。论文中报告了一些有害结果，如不适当的转诊和人员短缺加剧，这是由于担心承担负面后果（如指责、纪律处分或诉讼）所造成的。

**结论:**

有效实施孕产妇和/或围产期死亡监测和响应所需的条件包括：将该过程与诉讼和纪律程序独立开来；开展全面指导和培训；拥有足够资源以执行建议；以及提供确保安全学习的支持性监督。

## Резюме

**Цель:**

Понять опыт и восприятие людей, осуществляющих эпиднадзор за материнской и (или) перинатальной смертностью и ответные меры в странах с низким и средним уровнем дохода, а также механизмы, благодаря которым этот процесс может привести к намеченным результатам.

**Методы:**

В июне 2022 года в семи базах данных был проведен систематический поиск качественных исследований заинтересованных сторон, осуществляющих эпиднадзор за материнской и (или) перинатальной смертностью и ответные меры в странах с низким и средним уровнем дохода. Два рецензента, независимо друг от друга, отбирали статьи и оценивали их качество. Для выявления описательных тем использовался тематический синтез и реалистичный подход для понимания структуры контекста-механизма-результата.

**Результаты:**

Критериям включения соответствовали пятьдесят девять исследований. Благоприятные исходы (улучшение качества ухода или снижение смертности) подкреплялись функциональным циклом действий. В число механизмов эффективного эпиднадзора за смертностью и ответных мер входили обучение, внимательность и выполнение рекомендаций, которые мотивируют к дальнейшему участию. Ключевым контекстом для обеспечения эффективного эпиднадзора за смертностью и ответных мер являлась свободная от обвинений образовательная среда с хорошим руководством. Неудовлетворительные исходы (отсутствие улучшения ухода и снижения смертности, прекращение эпиднадзора за смертностью и ответных мер) стали результатом замкнутого круга занижения отчетных показателей, неточных данных, ненадлежащего анализа и рекомендаций, что привело к снижению мотивации и отстраненности. Сообщалось о некоторых неблагоприятных исходах, таких как ненадлежащие направления к врачам и усугубление нехватки персонала, что было вызвано страхом перед негативными последствиями, включая обвинения, дисциплинарные меры или судебные разбирательства.

**Вывод:**

В число необходимых условий для эффективного эпиднадзора за материнской и (или) перинатальной смертностью и ответных мер входят: отделение процесса от судебных разбирательств и дисциплинарных процедур; комплексные руководства и обучение; достаточные ресурсы для выполнения рекомендаций; поддерживающий надзор для обеспечения безопасного обучения.

## Introduction

**Fig. 1:**
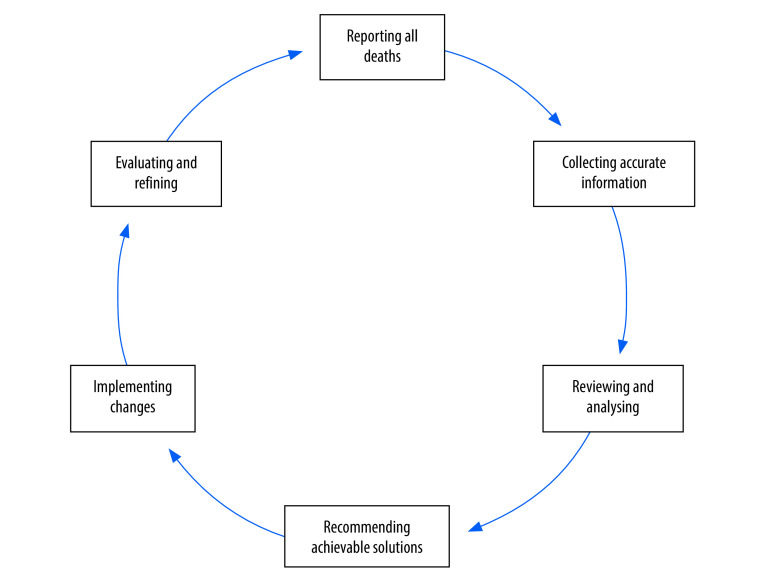
Maternal and perinatal death surveillance and response cycle

Many low- and middle-income countries are still far from attaining the sustainable development goals to reduce maternal and child mortality; one of the main obstacles is poor quality of health care.^1^ In 2004, the World Health Organization (WHO) recommended that all countries implement maternal death reviews,^2^ and in 2013 recommended all countries implement maternal death surveillance and response,^3^ to which perinatal deaths were added in 2016.^4^ Guidance on maternal and perinatal death surveillance and response was published in 2021.^5^ The existing programme theory, describing how the mortality audit cycle should function, is shown in Fig. 1 and Box 1.^2^^–^^5^

Box 1 **Programme theory for maternal and perinatal death surveillance and response**

**Identifying and reporting**

All maternal and perinatal deaths should be reported to produce valid statistics on mortality.

**Collecting information**

A truthful and complete account of the patient’s symptoms, treatment-seeking and management before their death should be obtained from verbal and/or social autopsy interviews, medical records and reports from health workers.

**Reviewing and analysing information**

The committee reviewing the account should reliably identify the cause of death and avoidable factors.

**Recommending solutions**

The committee should make effective recommendations to avoid recurrence of the same scenario.

**Implementing changes**

The recommendations made by the committee should be implemented.

**Evaluating and refining**

The implementation of the entire audit cycle should be monitored and, if necessary, changes should be made to achieve the desired goal of reducing maternal and perinatal mortality.

In a survey of low- and middle-income countries, 85% (88/103) had a national policy to review all maternal deaths.^6^ Most low- and middle-income countries that succeeded in reducing maternal and child mortality used some form of death reporting system to monitor progress, but only a minority used the full maternal and perinatal death surveillance and response cycle.^7^

Implementation of maternal and perinatal death surveillance and response in low- and middle-income countries is challenging because resources are more constrained than in high-income settings, but the opportunities to achieve a significant impact are greater. Maternal death reviews can reduce maternal mortality by up to 35% (odds ratio; OR: 0.65; 95% confidence interval, CI: 0.55–0.77) and perinatal death reviews have been associated with a 30% reduction in perinatal mortality (OR: 0.70; 95% CI: 0.62–0.79).^8^^–^^10^ However, these data from health facility studies represent a best-case scenario. When scaling up to the national level, the outcomes are more heterogeneous. For example, among 35 facilities that have been part of the South African Perinatal Problem Identification Programme for at least 5 years, perinatal mortality declined in four facilities, increased in five, and did not change in the remaining 26 facilities.^11^^,^^12^

The reasons for this heterogeneity in effectiveness are unclear. Several scoping reviews describe different maternal and perinatal death surveillance and response processes in sub-Saharan Africa and low- and middle-income countries, some with contradictory interpretations.^13^^–^^15^ While one review suggested that the most important mechanisms for accountability were disciplinary action, legal redress and social reprisals,^13^ another review reported that fear of blame and punitive approaches undermined the process.^14^ These reviews highlight the need for more research on death surveillance and review processes, the context in which they are conducted,^14^ and the subjective experiences of individuals implementing maternal and perinatal death surveillance and response in different settings.^15^ None of the previous reviews systematically analysed qualitative studies or took a realist approach to understanding why maternal and perinatal death surveillance and response systems achieve positive or negative outcomes in different contexts.

Therefore, in this systematic review, we aimed to understand the experiences of people implementing maternal and perinatal death surveillance and response in low- and middle-income countries. We sought to understand the mechanisms by which this process achieves (or fails to achieve) its intended outcomes, and the contexts that trigger these mechanisms.

## Methods

We conducted a systematic review of qualitative studies. The protocol was registered on PROSPERO (PROSPERO 2021 CRD42021271527).

### Literature search

We searched seven databases from their inception to June 2022: CINAHL, MEDLINE^®^, Embase^®^, ProQuest Dissertations and Theses, Global Index Medicus, Web of Science and Google Scholar. We used a pre-planned strategy including terms for maternal or perinatal death reviews from a Cochrane review^10^ and a search filter for qualitative studies (see strategy in first data repository).^16^

### Study selection

Two reviewers independently screened titles and abstracts against the inclusion criteria: studies using qualitative data collection and analysis methods, including participants who were involved in implementation of any part of the maternal and perinatal death surveillance and response process in low- and middle-income countries – including verbal and/or social autopsy when these involved investigation of maternal or perinatal deaths. We had no language restrictions. The reviewers then assessed the full text of the selected studies. We resolved disagreements by discussion with a third reviewer.

### Critical appraisal

One of the reviewers evaluated the quality of the included full-text articles using the Critical Appraisal Skills Programme tool for qualitative studies.^17^ The second reviewer independently evaluated a randomly selected 10% of the included articles; we found no significant disagreements.

### Data extraction and analysis

We imported studies into NVivo, version 12 (QSR International Inc., Burlington, MA, United States of America). We used a thematic synthesis approach:^18^ two authors developed a preliminary coding frame based on a sample of studies and refined this further by discussion. Higher-order categories of codes were deductive (barriers and enablers) but lower-order categories were developed inductively and iteratively from the data in the texts. We coded subsequent studies line by line, focusing on the results and discussion sections, and created new codes when considered necessary. We used the codes to develop descriptive themes. To develop higher-order analytical themes, we used a realist approach.^19^ We recoded the included articles specifically looking for contexts, mechanisms, outcomes and context–mechanism–outcome configurations.^19^^,^^20^ We used these configurations to construct flow diagrams showing causal links and to refine the programme theory for maternal and perinatal death surveillance and response.

## Results

### Studies included

**Fig. 2:**
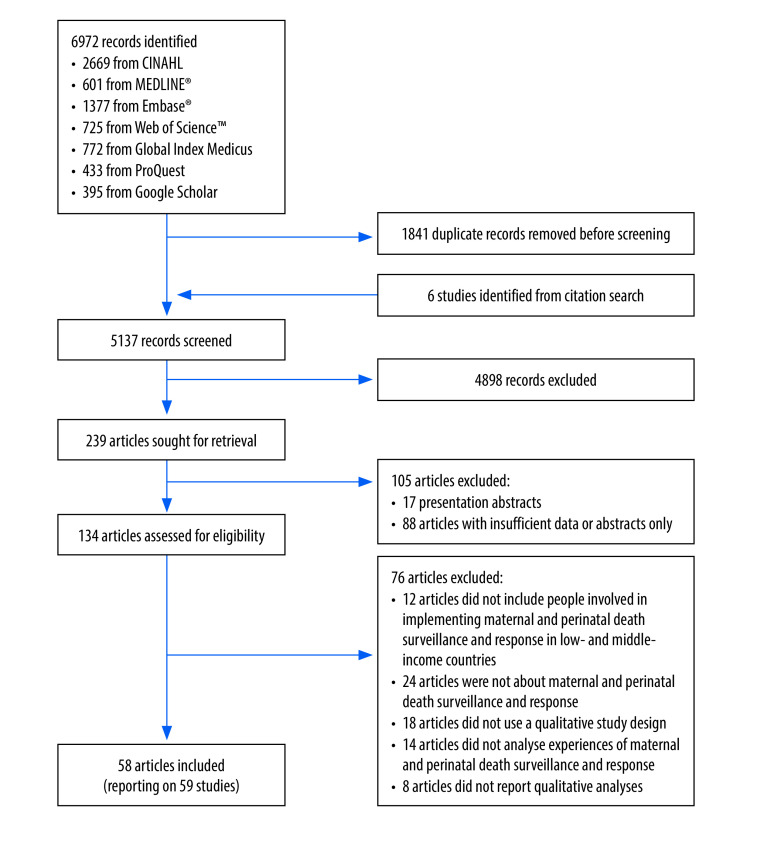
Flowchart of the selection of studies in the systematic review on maternal and perinatal death surveillance and response

The initial searches yielded a total of 5137 articles after removal of duplicates. After screening, we finally included 58 publications, reporting on 59 different studies (Fig. 2).^21^^–^^78^ These studies included over 1891 participants from 30 low- and middle-income countries, ranging from community members to health workers and national-level stakeholders involved in implementation of maternal death reviews or maternal and perinatal death surveillance and response.

Most studies (34/59) focused on maternal deaths (25 on maternal death reviews and nine on maternal death surveillance and response), 19 included both maternal and perinatal deaths, and six studies considered only perinatal or neonatal deaths (Table 1; available at: https://www.who.int/publications/journals/bulletin/). The overall effectiveness of the process was perceived as good (improved quality of care or reduced mortality) in 16 studies, inadequate in 21 studies and mixed in five studies; the perceived effectiveness was not reported in 17 studies. All studies were of sufficient quality (see details in the first data repository),^16^ although most did not adequately consider the relationship between the researcher and the participants.

**Table 1:** Studies on maternal and perinatal death surveillance and response included in the review

Study	Country, context	Type of death	Type of review	Perceived effectiveness of process	Study design	Data collection method	No. and type of participants	Type of analysis
Abbakar, 2021^21^	Sudan, national	Maternal	Maternal death surveillance and response	Inadequate	Qualitative	In-depth interviews	54 maternal death surveillance and response staff, doctors and midwives	Thematic analysis
Abebe, et al., 2017^22^	Ethiopia, national	Maternal	Maternal death surveillance and response	Successful	Qualitative	Individual and group interviews	69 frontline staff responsible for implementation of maternal death surveillance and response	Thematic content analysis
Aborigo et al., 2013^23^	Ghana, community	All	Verbal autopsy	Not specified	Qualitative	In-depth interviews	36 bereaved families, field staff, physicians and local leaders	Thematic analysis
Afayo, 2018^24^	Uganda, health facility	Maternal	Maternal death surveillance and response	Inadequate	Mixed methods	In-depth interviews	11 hospital staff and maternal death surveillance and response committee members	Thematic content analysis
Agaro et al., 2016^25^	Uganda, district health facility	Maternal and perinatal	Maternal and perinatal death surveillance and response	Inadequate	Mixed methods	Semi-structured interviews	76: 66 health workers and 10 key informants	Thematic content analysis
Armstrong et al., 2014^26^	United Republic of Tanzania, multiple levels	Maternal and perinatal	Maternal and perinatal death review	Inadequate	Qualitative	Document review and interviews	37: 20 hospital staff, 12 district or regional coordinators, 5 national experts	Adapted thematic analysis
Ayele et al., 2019^27^	Ethiopia, health facility and community	Maternal and perinatal	Maternal and perinatal death surveillance and response	Inadequate	Mixed methods	In-depth interviews and focus group discussions	25 women group leaders in 3 focus groups; 11 health managers in in-depth interviews	Thematic content analysis
Bakker et al., 2011^28^	Malawi, health facility (rural and district)	Maternal	Maternal death review	Successful	Qualitative	In-depth interviews, focus group discussions and observation	25 health workers	Not specified
Balogun & Musoke, 2014^29^	Sudan, national	Maternal	Maternal death review	Inadequate	Qualitative	In-depth interviews and focus group discussions	Medical and health stakeholders at the national, state and facility level in 12 in-depth interviews and 18 focus group discussions	Qualitative content analysis
Bandali et al., 2019^30^	Kenya, hospital and health centre	Maternal and perinatal	Maternal and perinatal death surveillance and response	Successful	Mixed methods	In-depth interviews and focus group discussions	5 health records information officers (interviews); maternal and perinatal death surveillance and response committee members (4 discussion groups)	Thematic analysis
Belizán et al., 2011^31^	South Africa, health facility	Perinatal	*Perinatal* *Problem Identification Programme*	Not specified	Qualitative	Focus group and workshop	48 clinicians and coordinators in the Perinatal Problem Identification Programme in 4 focus group discussions	Framework analysis using stages-of-change model
Boyi Hounsou et al., 2022^61^	Benin, health district	Maternal	Maternal death review	Inadequate	Mixed methods	Online group discussions	34 district medical officers in two online group discussions	Inductive thematic analysis
Biswas et al., 2014^33^	Bangladesh, community	Maternal, perinatal and neonatal	Maternal and perinatal death review	Successful	Mixed methods	In-depth interviews and focus group discussions	Health workers and community volunteers in 4 focus group discussions and 4 in-depth interviews	Thematic analysis
Biswas et al., 2015^34^	Bangladesh, health facility	Maternal, perinatal and neonatal	Maternal and perinatal death review	Successful	Qualitative	In-depth interviews, focus group discussions and document review	46 health workers implementing facility death review: 35 in in-depth interviews; 11 in focus group discussions	Thematic analysis
Biswas et al., 2015^32^	Bangladesh, community	Maternal, perinatal and neonatal	Verbal autopsy	Successful	Qualitative	In-depth interviews, focus group discussions and participant observation	Health-care providers: 3 focus group discussions, 6 in-depth interviews, 6 participant observations	Thematic analysis
Biswas et al., 2016^35^	Bangladesh, community	Maternal, perinatal and neonatal	Social autopsy	Successful	Qualitative	In-depth interviews, focus group discussions, observation and document review	Health inspectors in 9 focus group discussions; 18 health workers and 12 community members in in-depth interviews	Content and thematic analysis
Bvumbwe, 2019^62^	Malawi, health facility	Maternal	Maternal death review	Inadequate	Qualitative	In-depth interviews and focus group discussions	42 maternal death review committee members and 32 midwives: 4 focus group discussions with midwives; 4 focus group discussions with committee members; and 3 in-depth interviews with health zone technical officers	Thematic analysis
Cahyanti et al., 2021^36^	Indonesia, district health facility	Maternal	Maternal death review	Inadequate	Qualitative	Focus group discussions	29 district audit committee members in 4 focus group discussions	Thematic analysis
Chirwa et al., 2022^63^	Malawi, district hospital	Maternal	Maternal death review	Inadequate	Qualitative	In-depth interviews and focus group discussions	40 nurse midwives	Thematic content analysis
Combs Thorsen et al., 2014^37^	Malawi, urban health facility	Maternal	Maternal death review	Not specified	Mixed methods	Observation of participants of death review process	Observed data collection from bereaved family, health workers and medical records	Content analysis
Compaoré et al., 2022^64^	Ghana, health facility	Maternal	Maternal death review	Inadequate	Mixed methods	In-depth interviews	Health workers and managers	Not specified
Compaoré et al., 2022^65^	Liberia, county, health facility and community	Maternal and perinatal	Maternal and perinatal death surveillance and response	Inadequate	Mixed methods	In-depth interviews	County-level health personnel, health facility staff, community health workers	Not specified
Congo et al., 2017^38^, 2022^66^^,^^67^	Burkina Faso, regional and district hospital	Maternal	Maternal death review	Inadequate	Qualitative	In-depth interviews and document review	73 health workers in maternity, pharmacy and laboratory units, and staff in administration and management	Framework analysis
Dartey & Ganga-Limando, 2014^78^	Ghana, district hospital, regional referral hospital and teaching hospital	Maternal	Maternal death review	Successful	Qualitative	In-depth interviews	20 midwives involved in maternal death reviews	Thematic content analysis
Dartey, 2016 ^39^	Ghana, health centre, district hospital, regional referral hospital and teaching hospital	Maternal	Maternal death review	Successful	Mixed methods	In-depth interviews and focus group discussions	39 midwives involved in maternal death review: 18 in-depth interviews and 8 focus group discussions	Thematic content analysis
de Kok et al., 2017^40^	Nigeria, health facility	Maternal	Maternal death review	Not specified	Qualitative	Observation of review meetings	Audit review team	Conversation and discourse analysis
Diallo et al., 2022^68^	Burkina Faso, district hospital	Maternal	Maternal death review	Inadequate	Qualitative	In-depth interviews	9 midwives	Inductive thematic analysis
Dortonne et al., 2009^41^	Senegal and Mali, hospitals	Maternal	Maternal death review	Successful	Mixed methods	Questionnaires, checklist, interviews and document analyses	39: 23 maternal death audit committee members and 16 national-level leaders	Not specified
Dumont et al., 2009^42^	Senegal, health facility	Maternal	Maternal death review	Successful	Mixed methods	In-depth interviews, focus group discussions, participant observation and document reviews	Health workers (maternal health) in 3 focus group discussions and 9 in-depth interviews	Thematic analysis
Gao et al., 2009^43^	China, health facility, community	Maternal	Maternal death surveillance and response	Inadequate	Mixed methods	Interviews, field observations and review of reports and audits	18: 12 hospital leaders, 6 maternal and child health workers	Not specified
Hartsell, 2010^45^	United Republic of Tanzania, all levels (national, regional, district and health facility) including private and public facilities	Maternal	Maternal death review	Not specified	Descriptive qualitative case study	In-depth interviews, observation and document reviews	15 health workers involved in data management of maternal deaths and deliveries	Not specified
Hofman et al., 2014^46^	Nigeria, hospital	Maternal	Maternal death review	Not specified	Mixed methods	In-depth interviews	Members of the maternal death review committee of 11 hospitals (number not specified)	Thematic framework
Jati et al., 2019^69^	Indonesia, urban health facilities and local government in Semarang	Perinatal	Perinatal death surveillance and response	Not specified	Qualitative	Focus group discussions	20 local government officials and representatives of health facilities	
Jepkosgei et al., 2022^47^	Kenya, hospital	Neonatal	Neonatal death review	Not specified	Exploratory qualitative study	In-depth interviews, non-participant observation of morbidity and mortality meetings	Nurses and doctors: 17 in-depth interviews and 12 morbidity and mortality meetings	Thematic content analysis
Karimi et al., 2018^48^	Iran (Islamic Republic of), national, institutional (teaching universities) and health facility	Maternal	Maternal death surveillance and response	Successful	Qualitative	Review of documents and key informant interviews	15: 3 health ministry deputies, 10 medical university staff, 2 staff in obstetrics units of specialized hospitals	Thematic
Khader et al., 2020^70^	Jordan, health facility	Perinatal	Perinatal death audits	Not specified	Qualitative	Focus group discussions	Paediatricians, obstetricians, nurses, midwives in 16 focus group discussions	Thematic content analysis
Kinney et al., 2020^49^	Nigeria, United Republic of Tanzania, Zimbabwe, health facility	Maternal and perinatal	Maternal and perinatal death surveillance and response	Mixed	Mixed methods	Interviews and observation	41: 4 national stakeholders and 37 regional and district government health officials supporting maternal and perinatal death surveillance and response	Thematic content analysis
Kongnyuy et al., 2008^50^	Malawi, health facility	Maternal	Maternal death review	Successful	Mixed methods	Focus group discussions	60: maternal and neonatal health workers implementing the facility maternal death review and quality improvement team members	SWOT analysis
Kouanda et al., 2022^71^	Burundi, hospital	Maternal and perinatal	Maternal and perinatal death surveillance and response	Mixed	Qualitative	In-depth interviews	26 officials of the health ministry, hospital officers, officers of health regions and districts, and obstetricians and gynaecologists and midwives	Thematic analysis
Kouanda et al., 2022^72^	Chad, hospital (national, and district)	Maternal	Maternal death surveillance and response	Inadequate	Qualitative	In-depth interviews	25 officials at the central level, staff of technical and financial partners (WHO, UNFPA, UNICEF) and obstetricians and gynaecologists	Thematic analysis
Melberg et al., 2019^51^ and 2020^73^	Ethiopia, public health facility	Maternal	Maternal and perinatal death surveillance and response	Inadequate	Qualitative	In-depth interviews and observation	46: 11 primary caregivers who had experienced perinatal deaths, 5 men who had lost their partner to a maternal death, 4 health extension workers, 7 health workers in general and referral hospitals, 13 health workers in health centres, 6 health administrators responsible for implementation of maternal and perinatal death surveillance and response	Thematic content analysis
Muffler et al., 2007^52^	Morocco, health facility	Maternal	Maternal death review	Not specified	Mixed methods	In-depth interviews	56 implementers in the audit process	Systematic content analysis
Mukinda et al., 2021^74^	South Africa, health district and subdistrict	Maternal and perinatal	Maternal and perinatal death surveillance and response	Mixed	Descriptive qualitative case study	In-depth interviews and observation	45 frontline health managers and providers involved with maternal, perinatal, neonatal and child death surveillance and response	Thematic analysis
Muvuka, 2019^53^	Democratic Republic of the Congo, hospital and health facility	Maternal	Maternal death surveillance and response	Mixed	Qualitative	In-depth interviews, document review and observation of one maternal death review session	15 maternal death surveillance and response focal persons and members of maternal death review teams	Inductive thematic analysis
Nyamtema et al., 2010^54^	United Republic of Tanzania, hospital and health facility	Maternal and perinatal	Maternal and perinatal death review	Inadequate	Mixed methods	In-depth interviews and semi-structured questionnaire	59: 29 health managers and 30 health-care providers	Qualitative content analysis
Owolabi et al., 2014^55^	Malawi, health facility	Maternal	Maternal death review	Not specified	Mixed methods	In-depth interviews	8 individuals involved in implementing maternal death review	Thematic analysis
Patel et al., 2007^56^	India, community	Neonatal	Community neonatal death audits	Not specified	Qualitative	In-depth interviews and focus group discussions	Community members and family of the deceased in 3 in-depth interviews and 6 focus group discussions. Also included field staff from a subsequent study	Deductive thematic analysis
Richard, 2009^75^	Burkina Faso, urban district hospital	Maternal and perinatal	Maternal and perinatal death review	Not specified	Mixed methods	In-depth interviews	35 members of staff from maternity and surgical departments	Thematic analysis
Russell, 2022^76^	International, international expert consultation meeting	Maternal and perinatal	Maternal and perinatal death surveillance and response	Not specified	Qualitative	In-depth interviews and group interviews	55 health workers with experience in maternal and/or newborn health in humanitarian settings, and/or programmatic or research experience in maternal and perinatal death surveillance and response	Thematic analysis
Said et al., 2021^57^	United Republic of Tanzania, health facility	Maternal	Maternal death surveillance and response	Inadequate	Qualitative	In-depth interviews	60 involved in maternal death surveillance and response activities: 30 health providers in focus group discussions; 30 health managers in in-depth interviews	Inductive thematic analysis
Tayebwa et al., 2020^58^	Rwanda, health facility	Maternal and perinatal	Maternal and perinatal death surveillance and response	Not specified	Mixed methods	Desk reviews,in-depth interviews and observations	23: type not stated	Not specified
Upadhyaya et al., 2012^59^	India, district and peripheral health facility, community and/or village	Infant	Infant death review	Successful	Mixed methods	In-depth interviews and review of documents	38 health-care providers involved in programme activities	Content analysis
van Hamersveld et al., 2012^44^	United Republic of Tanzania, district hospital	Maternal and perinatal	Maternal and perinatal death review	Inadequate	Qualitative	Participant observation and in-depth interviews	23 health workers and managers	Inductive thematic analysis
WHO 2014^60^	India, all levels (national, regional, facility and community)	Maternal	Maternal death review	Successful	Mixed methods	Review of documents and reports, interviews and observations	Stakeholders at national, state and district levels	Not specified
	Indonesia, all levels (national, regional, facility and community)	Maternal	Maternal death review	Mixed	Mixed methods	Review of documents and reports, and interviews	Informants from the health ministry, district health office, hospitals and health centres	Not specified
	Sri Lanka, national	Maternal	Maternal death review	Successful	Mixed methods	Stakeholder workshop and in-depth interviews	20 former secretaries of health, former directors of the Family Health Bureau, provincial administrators, clinicians, representatives of professional colleges, national programme managers and representatives from international NGOs	Not specified
	Nepal, national	Maternal	Maternal death review	Not specified	Mixed methods	Document review, in-depth interviews and stakeholder workshop	27: 16 doctors, 4 staff nurses, 5 medical recorders and 2 programme managers from 10 hospitals	Not specified
	Myanmar, national	Maternal	Maternal death review	Not specified	Mixed methods	In-depth interviews	10–12 participants from 10 townships including township medical officer, obstetricians, township health nurse, station medical officers, focal persons of a rural health centre, and midwives	Not specified
Yameogo et al., 2022^77^	Burkina Faso, health district (urban and rural)	Maternal	Maternal death surveillance and response	Inadequate	Qualitative	In-depth interviews	23: 3 technical and financial partners, 2 central level managers, 2 regional health directors, 4 district management team members, 8 health-care providers and 4 community health workers	Thematic analysis

NGO: nongovernmental organization; SWOT: strengths, weaknesses, opportunities and threats; UNFPA: United Nations Population Fund; UNICEF: United Nations Children’s Fund; WHO: World Health Organization.

**Fig. 3:**
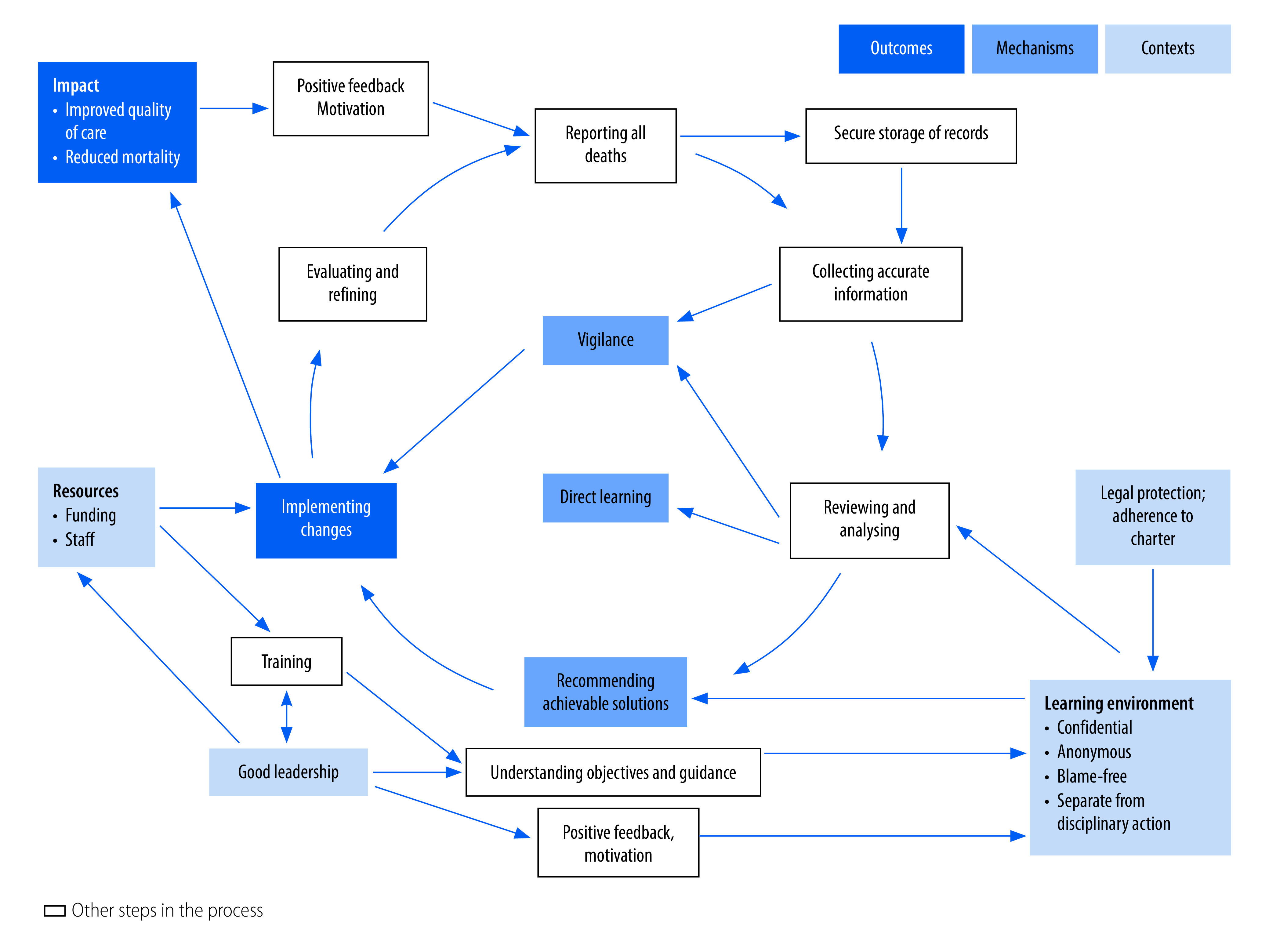
Action cycle of a functional maternal and perinatal death surveillance and response process

**Fig. 4:**
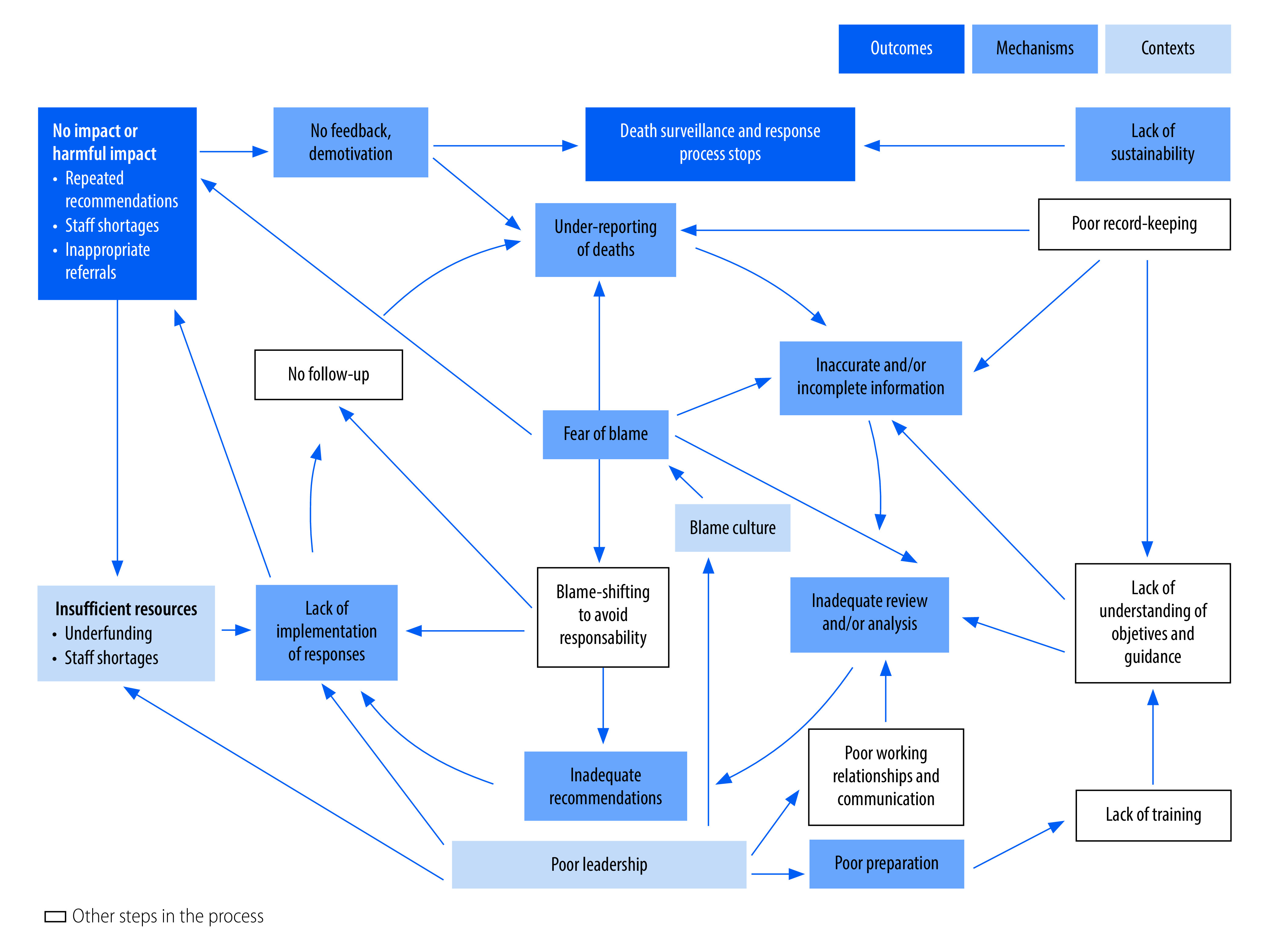
Vicious cycle of a dysfunctional maternal and perinatal death surveillance and response process

Two overarching programme theories emerged from our review of the studies: (i) a refined version of the classic action cycle, which explains how functional maternal and perinatal death surveillance and response systems reduce maternal and perinatal mortality (Fig. 3 and Table 2; full table in the second data repository);^79^ and (ii) the vicious cycle, which explains how dysfunctional systems can fail to achieve their intended objectives, or worse, lead to unintended harmful outcomes (Fig. 4 and Table 3; full table in the second data repository).^79^

**Table 2:** Mechanisms and contexts underlying functional maternal and perinatal death surveillance and response systems

Key mechanisms driving good outcomes	Key contexts that enable these mechanisms to operate	Examples, study and country^a^
Preparing for implementation	Supportive national policy	Biswas et al., Bangladesh^33^
	Clear guidelines	Biswas et al., Bangladesh^35^
	Comprehensive training of all stakeholders	Agaro et al., Uganda^25^ Bandali et al., Kenya^30^
	Good, committed and supportive leadership and drivers at all levels	Belizán et al., South Africa^31^ Dortonne et al., Senegal and Mali^41^
	Blame-free learning environment	Jepkosgei et al., Kenya^47^
Implementing comprehensive death reporting	Clear responsibilities	Biswas et al., Bangladesh^34^
	Clear lines of communication	Said et al., United Republic of Tanzania^57^
Collecting accurate information	Clear, accurate documentation	Biswas et al., Bangladesh^34^
	Secure storage of records	Muvuka, Democratic Republic of the Congo^53^
	User-friendly forms	WHO, Nepal^60^
	Appropriate timing to interview families	Aborigo et al., Ghana^23^
	Appropriate person to interview families	Biswas et al., Bangladesh^33^ Dumont et al., Senegal^42^
	Validation of data	Aborigo et al., Ghana^23^ Biswas et al., Bangladesh^32^
Learning through participation in reflective review and analysis	Inclusive multidisciplinary review committee with key stakeholders, working as a team	Bandali et al., Kenya^30^ Muvuka, Democratic Republic of the Congo^53^
	Clear communication about meetings	Congo et al., Burkina Faso^38^
	Meetings embedded into routine work responsibilities	Belizán et al., South Africa^31^ Muvuka, Democratic Republic of the Congo^53^
	Good attendance at review meetings	Bakker et al., Malawi^28^
	Refreshments for staff at meetings	Jepkosgei et al., Kenya^47^
	Skilled chairing to ensure the discussion is confidential, anonymous, blame-free (but with accountability), participatory, focused and time-efficient, and a useful learning experience for all involved	Armstrong et al., United Republic of Tanzania^26^ de Kok et al., Nigeria^40^ Jepkosgei et al., Kenya^47^
	Structured discussion	Jepkosgei et al., Kenya^47^
	Evaluation of care against accepted standards	Cahyanti et al., Indonesia^36^ Kongnyuy et al., Malawi^50^
Recommending achievable solutions	Focus on achievable goals	Bandali et al., Kenya^30^
	Involvement of the people who will need to implement the solutions	Bandali et al., Kenya^30^ Biswas et al., Bangladesh^35^ Kinney et al., Zimbabwe^49^
	Clear assignment of responsibility for each recommendation	Belizán et al., South Africa^31^ van Hamersveld et al., United Republic of Tanzania^44^
	Documentation of the recommendations and dissemination to all relevant stakeholders	Bandali et al., Kenya^30^ Muvuka, Democratic Republic of the Congo^53^
Implementing changes	Changes that can be incorporated within existing budget and workplan; sufficient resources to implement them	Abebe et al., Ethiopia^22^ Agaro et al., Uganda^25^
	Direct learning from the review	Biswas et al., Bangladesh^35^ Said et al., United Republic of Tanzania^57^
	Emotional impact of the review	Dartey, Ghana^39^ Richard et al., Burkina Faso^75^
	Vigilance because of the review process	van Hamersveld et al., United Republic of Tanzania^44^ Muvuka, Democratic Republic of the Congo^53^
	Communities motivated to raise funds	Hofman & Mohammed, Nigeria^46^ WHO, Myanmar^60^
	Recommendations transmitted and implemented at national level	Abbakar, Sudan^21^
	Follow-up of implementation	Armstrong et al., United Republic of Tanzania^26^ Bandali et al., Kenya^30^ Mukinda et al., South Africa^74^
Evaluating and refining	Positive feedback	Bandali et al., Kenya^30^ Muffler et al., Morocco^52^ WHO, South-East Asia^60^
	Supervision and mentoring, external champions and facilitators	Belizán et al., South Africa^31^ Bandali et al., Kenya^30^ Dortonne et al., Mali and Senegal^41^

WHO: World Health Organization.

^a^ See second data repository for full table with quotations and comments.^79^

**Table 3:** Contexts and mechanisms underlying dysfunctional maternal and perinatal death surveillance and response systems

Key mechanisms driving poor outcomes	Key contexts that enable mechanisms to operate	Examples, study and country^a^
Fear of blame (at all levels)	Political pressure to reduce maternal deaths	Melberg et al., Ethiopia^51^
	Punitive environment	Abbakar, Sudan^21^ Abebe et al., Ethiopia^22^ Combs Thorsen et al., Malawi^37^ Melberg et al., Ethiopia^73^
	Increasing litigation against health workers	Gao et al., China^43^ Melberg et al., Ethiopia^73^
	Blame culture: maternal and perinatal death surveillance and response process is not separated from litigation and disciplinary process	Cahyanti et al., Indonesia^36^ Karimi et al., Iran (Islamic Republic of)^48^ Muvuka, Democratic Republic of the Congo^53^
Inadequate preparation	Guidelines insufficient or non-existent	Abebe et al., Ethiopia^22^ Muvuka, Democratic Republic of the Congo^53^
	Staff unaware of guidelines	Cahyanti et al., Indonesia^36^ Said et al., United Republic of Tanzania^57^
	Lack of training	Abebe et al., Ethiopia^22^ Congo et al., Burkina Faso^38^ Said et al., United Republic of Tanzania^57^
	Poor leadership: no support for staff	Afayo, Uganda^24^ Muffler et al., Morocco^52^
	Vertical process, not integrated	Balogun & Musoke, Sudan^29^ Hartsell, United Republic of Tanzania^45^
Under-reporting of deaths	Fear of blame	Abbakar, Sudan^21^ Melberg et al., Ethiopia^51^ Muvuka, Democratic Republic of the Congo^53^
	Political pressure	Khader et al., Jordan^70^, Melberg et al., Ethiopia^51^
	Social stigma and cultural beliefs	Biswas et al., Bangladesh^33^ Muvuka, Democratic Republic of the Congo^53^
	No mandatory reporting for out-of-hospital deaths	Dumont et al., Senegal^42^ Muvuka, Democratic Republic of the Congo^53^
Inaccurate or incomplete information	Fear of blame: concealing or falsifying information	Agaro et al., Uganda^25^ Muvuka, Democratic Republic of the Congo^53^ Said et al., United Republic of Tanzania^57^
	Staff lack of understanding of purpose	Kinney et al., Nigeria^49^
	Poor record-keeping	Dumont et al., Senegal^42^ Muvuka, Democratic Republic of the Congo^53^
	Resource shortages: insufficient time to collect data	Hartsell, United Republic of Tanzania^45^
	Data collection forms too long and/or complex and/or unavailable	WHO, Myanmar^60^
Inadequate review	Inaccurate and/or insufficient information impeding review process	Gao et al., China^43^ Owolabi et al., Malawi^55^
	Key stakeholders not involved or invited	Abbakar, Sudan^21^ Dumont et al., Senegal^42^ Gao et al., China^43^ Jepkosgei et al., Kenya^47^
	Non-attendance of review committee members because of staff shortages, workload, competing priorities, poor communication or demotivation	Afayo, Uganda^24^ Kinney et al., United Republic of Tanzania^49^ Muvuka, Democratic Republic of the Congo^53^ Congo et al., Burkina Faso^67^ ,van Hamersveld et al., United Republic of Tanzania^44^
	Lack of incentives to participate	Afayo, Uganda^24^ Agaro et al., Uganda^25^
	Ineffective participation of members because of demotivation and/or hierarchy	Armstrong et al., United Republic of Tanzania^26^ Cahyanti et al., Indonesia^36^ de Kok et al., Nigeria^40^ Richard et al., Burkina Faso^75^
	Lack of confidentiality	Muvuka, Democratic Republic of the Congo^53^ Congo et al., Burkina Faso^67^
	Fear of blame	Jepkosgei et al., Kenya^47^ Muffler et al., Morocco^52^
	Blame-shifting and/or avoiding responsibility	Jepkosgei et al., Kenya^47^ Melberg et al., Ethiopia^51^
Inadequate recommendations	Poor chairing	Jepkosgei et al., Kenya^47^
	Lack of focus during meetings	de Kok et al., Nigeria^40^ Hartsell, United Republic of Tanzania^45^ WHO, Indonesia^60^
	Blame-shifting and/or avoiding responsibility	Armstrong et al., United Republic of Tanzania^26^ Cahyanti et al., Indonesia^36^ Gao et al., China^43^
Inadequate implementation	Recommendations not actionable	Muvuka, Democratic Republic of the Congo^53^
	Key stakeholders (responsible for implementation) absent from meetings	Nyamtema et al., United Republic of Tanzania^54^ WHO, India^60^
	Unclear responsibility and/or accountability	Armstrong et al., United Republic of Tanzania^26^
	Avoidance of responsibility	Balogun & Musoke, Sudan^29^ Cahyanti et al., Indonesia^36^
	Insufficient resources to allow implementation	Agaro et al., Uganda^25^ Cahyanti et al., Indonesia^36^ Karimi et al., Iran (Islamic Republic of)^48^
	Lack of feedback and/or dissemination of recommendations	Kouanda et al., Chad^72^
	Lack of follow-up; no feedback or incentive to implement	Jepkosgei et al., Kenya^47^
Demotivation, disengagement, discontinuation	Demotivation of participants because of lack of implementation or positive feedback	Agaro et al., Uganda^25^ Muffler et al., Morocco^52^ Nyamtema et al., United Republic of Tanzania^54^
	Lack of supportive supervision	Agaro et al., Uganda^25^ Muvuka, Democratic Republic of the Congo^53^
Unintended harmful consequences	Exacerbation of staff shortages	Bakker et al., Malawi^28^ Kinney et al., United Republic of Tanzania^49^
	Defensive practice, inappropriate referrals	Melberg et al., Ethiopia^51^
Unsustainable process	Over-dependence on foreign aid	Congo et al., Burkina Faso^38^ Hofman & Mohammed, Nigeria^46^ Said et al., United Republic of Tanzania^57^ Kouanda et al., Chad^72^
	Frequent staff turnover and lack of handover and training	Abebe et al., Ethiopia^22^ Hofman & Mohammed, Nigeria^46^
	Over-dependence on one person	Abbakar, Sudan^21^ van Hamersveld et al., United Republic of Tanzania^44^

WHO: World Health Organization.

^a^ See second data repository for full table with quotations and comments.^79^

### Action cycle

#### Outcomes

Successful outcomes of maternal and perinatal death surveillance and response included implementation of positive changes, especially at the facility level, such as improvements in quality of care, behavioural changes and targeted actions to address specific issues. Two studies^41^^,^^50^ were linked to quantitative studies^8^^,^^80^ demonstrating reductions in mortality.

#### Mechanisms

Three key mechanisms led to implementation of positive change.

##### Implementation of recommendations

Formulation and implementation of effective recommendations are commonly assumed to be the only mechanism of action for maternal and perinatal death surveillance and response.^4^ They are underpinned by a relatively complicated chain of events (Fig. 3 and Table 2). Most examples of effective responses were targeted actions implemented in individual facilities.^25^ Although WHO guidelines recommend that aggregated data be analysed at district and national levels to identify, recommend and implement higher-level solutions,^6^ documented examples of these actions were rare.^21^

##### Learning from case discussions

Learning from mistakes was a powerful behaviour-change mechanism mentioned by several respondents and was facilitated by a learning environment in the facility^47^ and community-based review meetings.^35^ Behaviour change was also motivated by the emotional experience of hearing the stories about the maternal and perinatal deaths and how these cases had been (mis)managed.^39^^,^^62^^,^^75^

##### Increased vigilance

This learning, and the review process itself, were reported to make health workers more vigilant in their daily practice, because they knew that if a patient died, their actions and records would be reviewed.^44^^,^^53^^,^^75^

#### Contexts

Underpinning these mechanisms is a learning environment (Fig. 3), where people feel safe to honestly report deaths, disclose accurate information and openly discuss the cases, including any mistakes in their management.^47^^,^^53^^,^^56^^,^^74^ Learning environments assure confidentiality, anonymity and separation from blame or any disciplinary process. Although several respondents recommended legal protection at the national level to prevent data from maternal and perinatal death surveillance and response being used in litigation, only South Africa had enacted this protection which “has been ratified by relevant judicial bodies.”^81^

In the absence of such legal protection, the next best context was an audit charter; members of the maternal and perinatal death surveillance and response committee were required to sign this charter to indicate their commitment to the principles of good conduct of clinical audit, including confidentiality, before participating in any session.^38^^,^^75^ Good leadership and chairing of meetings at the facility level also create a safe space for open discussion (Fig. 3 and Table 2).^40^ Adequate resources enable implementation of the process and of recommendations.

### Vicious cycle

In contrast, many studies reported elements of a vicious cycle resulting in dysfunctional death surveillance and response (Fig. 4 and Table 3).

#### Outcomes

The commonest negative outcome was simply the lack of any change.^49^^,^^77^ In some cases, the maternal and perinatal death surveillance and response process stopped.^72^ Two studies reported on the maternal and perinatal death review process in the same urban district hospital in Burkina Faso in 2004–2005^75^ and 2015–2016.^77^ Although this was one of the pioneer hospitals, in the second study an informant from the district level reported, “I know the team is there, but I don’t believe that this committee ever has a session.”^77^

More worryingly, a few studies reported harmful outcomes. First, staff shortages could be worsened as staff became afraid to work on the labour ward,^28^^,^^62^ some took several weeks off work after an upsetting review^73^ and junior doctors were deterred from choosing obstetrics as a career.^73^ Second, some staff practised defensive medicine such as inappropriate referral of unstable patients at high risk of death.^51^^,^^73^ Third, an extreme example given was refusal of admission to referral facilities of women who seemed likely to die, possibly to avoid damaging mortality statistics.^76^ Fourth, serious repercussions were reported for a woman who had complained that a midwife had treated her harshly; the midwife recognized herself in the audit session and complained to the woman’s parents.^75^

#### Mechanisms

Fear of blame (and of negative consequences such as disciplinary action or litigation) was the most pervasive mechanism. This fear inhibited learning and participation, and led to disengagement from the maternal and perinatal death surveillance and response process at all stages, which resulted in under-reporting, inaccurate data, inadequate participation in reviews, inadequate formulation of solutions and avoidance of responsibility. Fear of blame usually resulted from insufficient confidentiality or anonymity, and the death review process not being separated from disciplinary procedures.^76^ Telling participants that the process was blame-free was insufficient to allay fears when senior managers were present who would also be in charge of disciplinary procedures^53^^,^^76^ or when litigation against health workers was increasing.^73^

Inadequate preparation enabled the blame culture to persist as staff were unsure how to implement maternal and perinatal death surveillance and response.^22^ Many references were made to: inadequate or unavailable guidance; lack of training; poor leadership; charters not being signed;^38^ and maternal and perinatal death surveillance and response being structured as a separate vertical programme rather than being integrated with other public health systems.^29^^,^^45^

Under-reporting of deaths was often due to fear of blame or other negative consequences, such as reduced funding,^21^^,^^53^^,^^73^^,^^76^ but also resulted from social stigma,^33^ cultural beliefs, non-mandatory reporting^53^ and political pressure.^51^^,^^72^^,^^73^

Inaccurate and/or incomplete information undermines the review process. Although poor record-keeping was common,^42^^,^^53^ several reports noted deliberate falsification of records^25^^,^^57^^,^^70^^,^^73^ or misclassification of deaths^70^^,^^76^ to avoid blame or reputational damage. Sometimes staff did not collect the information because they simply did not have time^45^ or the correct forms,^60^ or did not understand the purpose of maternal and perinatal death surveillance and response.^49^

Inadequate review was the inevitable consequence of inaccurate information: “it is essentially garbage in, garbage out.”^55^ Reviews could also fail if: the committee did not include all necessary stakeholders; some key stakeholders did not attend; stakeholders attended but felt unable to participate because of disengagement or hierarchical relationships; or stakeholders feared blame or attempted to shift blame to others.^26^^,^^36^^,^^40^

Inadequate recommendations result from inadequate review. Poor chairing, lack of focus in review meetings and blame-shifting^26^^,^^36^^,^^43^ also impaired the formulation of effective recommendations.^40^ Sometimes meetings focused on accurately determining the cause of death at the expense of formulating effective recommendations.^45^

Non-implementation of recommendations was inevitable if they were unachievable. Furthermore, implementation rarely happened if: responsibility for implementation was unclear;^44^ the individuals responsible for implementation were not involved in the review;^21^^,^^38^^,^^54^^,^^60^ recommendations were not fed back to those responsible for implementation;^30^^,^^44^ implementers avoided taking responsibility;^40^^,^^43^ or no mechanism was in place to follow up on implementation.^76^^,^^77^ Insufficient resources also prevented implementation.^25^^,^^36^^,^^48^^,^^72^

Demotivation and disengagement resulted from non-implementation and the perception that the process was not achieving its intended aim.^25^^,^^52^^,^^54^ The lack of any incentives was also demotivating.^24^^,^^25^^,^^76^

Lack of sustainability resulted from over-dependence on foreign aid,^38^^,^^46^^,^^72^ or on a small number of staff.^21^ If no team or mechanism existed for training new staff, the process would stop when key staff were absent or left, which was common given high staff turnover in many settings.

#### Contexts

Three key contexts triggered the mechanisms leading to dysfunctional maternal and perinatal death surveillance and response. First, a blame culture heightens fear of blame, which was widely reported in health workers and families being questioned about a death. This problem was exacerbated in countries under an authoritarian system, where confidentiality was not guaranteed^75^ and the maternal and perinatal death surveillance and response process was not separated from litigation or disciplinary procedures,^51^ where families had no avenues for complaining apart from litigation,^73^ and where health workers could be detained by the police after maternal or child deaths.^22^^,^^73^^,^^82^ Paradoxically, high-level political commitment to reducing maternal mortality sometimes resulted in pressure on health workers not to report deaths.^51^^,^^72^^,^^73^

Second, insufficient resources prevented: adequate preparation for maternal and perinatal death surveillance and response; adequate data collection; convening of review meetings; and implementation of recommendations.^60^^,^^63^ Staff shortages meant that key stakeholders could not leave clinical duties to complete investigations or attend meetings^34^^,^^44^^,^^50^^,^^53^ and also that anonymity was not possible in review meetings.^67^ In some cases, sufficient forms were not available.^60^ Staff were often expected to attend meetings during lunch breaks or after work, but were reluctant to do so if no refreshments or financial compensation were provided.^25^ Lack of any budget for maternal and perinatal death surveillance and response also made it difficult to implement many recommendations;^44^ for example buying new equipment or holding community meetings.

Third, poor leadership at facility, district or national levels perpetuated unfavourable environments and behaviour, including: the blame culture,^63^ a general lack of commitment to maternal and perinatal death surveillance and response,^54^^,^^72^ under-resourcing, frequent staff turnover, poor preparation for maternal and perinatal death surveillance and response, insufficient communication, poor chairing of surveillance and response meetings,^52^ non-implementation and follow-up of recommendations, and general demotivation.^42^

## Discussion

We found 59 qualitative studies investigating implementation of maternal and perinatal death surveillance and response in low- and middle-income countries. To achieve a functional action cycle with positive outcomes, such as reduced mortality and improved quality of care, a blame-free learning environment needs to be nurtured, clearly separated from litigation and disciplinary processes. Although WHO guidelines state that a mortality audit “is not a solution in itself,”^4^ several studies found that a learning environment enables not only the formulation of achievable recommendations, but also direct learning from the process and a healthy vigilance regarding quality of care. Good outcomes motivate staff to remain engaged, making the process sustainable.

In stark contrast, maternal and perinatal death surveillance and response often became a dysfunctional vicious cycle in the context of a blame culture, poor leadership and insufficient resources. Fear of blame inhibits all steps of the surveillance and response cycle. This fear not only inhibits intended outcomes but can also provoke harmful outcomes such as falsification of information, worsened staff shortages, inappropriate referrals or even the refusal to accept referrals, with the intention of avoiding responsibility. Our findings contradict the conclusions of the 2016 study that reported disciplinary action, legal redress and social reprisals were the most important mechanisms for accountability:^13^ we found that disciplinary action, litigation and social reprisals were likely to result in disengagement, lack of learning and negative outcomes.

While the literature search was comprehensive and the realist approach provided a useful framework for understanding causal pathways, the maternal and perinatal death surveillance and response process is cyclical rather than linear and a particular issue could be a context, a mechanism or an outcome at different points in the cycle. While other study types may also contain useful information, we only included qualitative studies because we were interested in the subjective experiences of those participating in maternal and perinatal death surveillance and response. However, social desirability bias is likely to be an important weakness of any research in contexts where freedom of speech is limited and a fear of blame exists, both of which may prevent participants from being completely open and honest about their experiences.^51^ Nevertheless, our review included several articles giving candid accounts of dysfunctional maternal and perinatal death surveillance and response processes in several settings. As the bias is likely to favour positive accounts, the reality could be worse than has been reported.

Most studies did not adequately consider the relationship between researchers and interviewees, and it is likely that this relationship influenced reported perceptions of the success, or failure, of the maternal and perinatal death surveillance and response process. Furthermore, implementation of maternal and perinatal death surveillance and response may have both positive and negative aspects in a single country or study.

Our results have implications for policy and practice. First, it is imperative to ensure that necessary preparations have been made before attempting to implement a maternal and perinatal death surveillance and response process. The essential conditions to ensure an effective process are good leadership, willingness and ability to provide a safe, blame-free learning environment and sufficient resources to support the surveillance and response process and implementation of its recommendations. In the context of a blame culture (including litigation and disciplinary procedures), poor leadership and insufficient resources, the process could do more harm than good. Turning a vicious cycle into an action cycle can be more difficult than starting the whole process from scratch, because fear of blame can persist for a long time.^53^

Second, direct learning from review meetings has been ignored as an important mechanism by many implementers. Thus, participatory review meetings on site and involving as many relevant staff as possible are likely to be more effective at promoting positive behaviour change than remote committee meetings with only a small number of participants.

Third, to evaluate maternal and perinatal death surveillance and response, it is important to assess not only the level of implementation of recommendations, but also whether participants are learning from the process, changing their own practice and seeing positive changes. Monitoring for possible adverse events of the process is also important, such as inappropriate referrals or worsening staff shortages. Monitoring and evaluation focusing on death reporting and cause of death classification may detract from the response component to improve outcomes.

Fourth, an adaptable toolbox of strategies to improve implementation of maternal and perinatal death surveillance and response would be valuable, based on experiences identified through this review as well as behaviour-change theory. 

Our findings revealed priorities for future research. First, an intervention to improve implementation of maternal and perinatal death surveillance and response could be co-created with teams already conducting this process in low-income contexts, based on their experience and findings from this review. Scarce resources should not be a barrier to implementation, as several examples of effective review processes in low- and middle-income countries exist.^8^^–^^10^ A behavioural science approach should be taken to planning and optimizing the intervention, for example using the person-based approach,^83^ with members of death review committees in different settings. Of particular importance would be to evaluate whether such an intervention can shift a vicious cycle into a positive action cycle.

Second, more research is needed to understand how to achieve the optimal balance between a blame-free anonymous process, while maintaining accountability.^47^ Although WHO has suggested high-level strategies to minimize the blame culture,^5^^,^^84^ challenges exist because a completely blame-free, anonymous process may also remove accountability and responsibility for implementing actions,^73^ while a focus on accountability may instil fear of blame.^73^ Completely removing blame from the maternal and perinatal death surveillance and response process is almost impossible, because negligence will be uncovered and will need to be tackled.^57^ Although disciplinary procedures should be kept separate from maternal and perinatal death surveillance and response, in practice this separation may be impossible to achieve in district hospitals and communities where the head of the maternity unit is probably responsible for both disciplinary procedures and the surveillance and response process. A certain level of accountability and vigilance is one of the key mechanisms for a maternal and perinatal death surveillance and response system to achieve its objectives. A sensitive, inclusive death review process could provide a way to address concerns of bereaved families and sensitively inform them about their loss; this approach is important to explore, as it could reduce conflict and unjustified blame of individual health workers.^70^^,^^73^

In conclusion, maternal and perinatal death surveillance and response can be an effective behaviour-change quality-improvement intervention even in low- and middle-income settings with limited resources, provided the process is conducted in a largely blame-free learning environment, supported by good leadership and sufficient resources.
